# Consensus on the Definition of Advanced Parkinson's Disease: A Neurologists-Based Delphi Study (CEPA Study)

**DOI:** 10.1155/2017/4047392

**Published:** 2017-01-23

**Authors:** Maria-Rosario Luquin, Jaime Kulisevsky, Pablo Martinez-Martin, Pablo Mir, Eduardo S. Tolosa

**Affiliations:** ^1^Department of Neurology, Clínica Universidad de Navarra, Universidad de Navarra, Pamplona, Spain; ^2^Department of Neurology, Movement Disorders Unit, Hospital de la Santa Creu i Sant Pau (IIB Sant Pau), Universitat Autònoma de Barcelona, Universitat Oberta de Catalunya (UOC), Barcelona, Spain; ^3^Centro de Investigación Biomédica en Red sobre Enfermedades Neurodegenerativas (CIBERNED), Madrid, Spain; ^4^Area of Applied Research, National Center of Epidemiology, Carlos III Institute of Health, Madrid, Spain; ^5^Unidad de Trastornos del Movimiento, Servicio de Neurología y Neurofisiología Clínica, Instituto de Biomedicina de Sevilla, Hospital Universitario Virgen del Rocío/CSIC/Universidad de Sevilla, Seville, Spain; ^6^Neurology Service, Hospital Clínic, IDIBAPS, Universitat de Barcelona, Barcelona, Spain

## Abstract

To date, no consensus exists on the key factors for diagnosing advanced Parkinson disease (APD). To obtain consensus on the definition of APD, we performed a prospective, multicenter, Spanish nationwide, 3-round Delphi study (CEPA study). An ad hoc questionnaire was designed with 33 questions concerning the relevance of several clinical features for APD diagnosis. In the first-round, 240 neurologists of the Spanish Movement Disorders Group participated in the study. The results obtained were incorporated into the questionnaire and both, results and questionnaire, were sent out to and fulfilled by 26 experts in Movement Disorders. Review of results from the second-round led to a classification of symptoms as indicative of “definitive,” “probable,” and “possible” APD. This classification was confirmed by 149 previous participating neurologists in a third-round, where 92% completely or very much agreed with the classification. Definitive symptoms of APD included disability requiring help for the activities of daily living, presence of motor fluctuations with limitations to perform basic activities of daily living without help, severe dysphagia, recurrent falls, and dementia. These results will help neurologists to identify some key factors in APD diagnosis, thus allowing users to categorize the patients for a homogeneous recognition of this condition.

## 1. Introduction

Parkinson disease (PD) is the second most common age-related neurodegenerative disorder after Alzheimer's disease, affecting nearly 1% of the population over 60 years and 5% in subjects up to 85 years [1, 2], with high health, social, and economic impact [3].

While currently effective antiparkinsonian drugs are available, allowing patients to have an acceptable functional capacity during the early years of PD, as time goes by, motor and functional deterioration develop, partly due to the presence of motor and nonmotor complications, highly influencing patients' quality of life. At this stage, the conventional medication is unable to provide an adequate clinical control [4–6] and the term advanced PD is frequently used (APD).

However, the term APD is still controversial and is variably applied to patients with long disease duration or with motor fluctuations and severe or moderate dyskinesia, with disorders of gait and equilibrium, or with cognitive impairment or neuropsychiatric symptoms [4, 7, 8]. Many of these symptoms do not improve with conventional therapies, although for motor fluctuations and dyskinesias there are alternative treatments, so-called “advanced therapies,” including deep brain stimulation (DBS), continuous subcutaneous apomorphine infusion, or the infusion of levodopa/carbidopa intestinal gel [9], to which the majority of patients can have a satisfactory response. Therefore, it is of interest to know the patients' clinical characteristics that can define APD and that make these patients eligible for advanced therapies.

In this context, the primary objective of this study was to reach a consensus on the definition of APD using three round questionnaires with Delphi methodology.

## 2. Methods

### 2.1. Study Design

This multicenter study was performed using Delphi method [10–12]. The study protocol was approved by the Ethics Committee of Hospital Clínic i Provincial de Barcelona (Spain) in May 2013.

### 2.2. Scientific Committee

Five neurologists from 5 different Spanish centers, experts in Movement Disorders and PD, formed the Scientific Committee. After a careful review of the literature [13], a questionnaire reflecting some of the proposed features defining APD (clinical characteristics and treatment) was created. The Scientific Committee validated the methodology, study design, and study protocol and prepared the questionnaire, analyzed the results of the two first rounds, and added the statistical analysis from the first-round to the second questionnaire. Based on the results of the two rounds, the Scientific Committee elaborated a list of clinical characteristics of APD, which was again sent to all participants (third-round) to reach a final consensus.

### 2.3. Questionnaires

The first-round questionnaire included a total of 33 items with a 0–4 scale: 0 (no determinant), 1 (little), 2 (some), 3 (sufficiently), and 4 (absolutely) (available at http://cepa.medynet.com/).

The selected questions assessed the significance of 7 different factors in the diagnosis and definition of APD (general characteristics of the disease, type and level of disability, presence and severity of motor fluctuations and dyskinesia induced by dopaminergic drugs, occurrence and severity of motor and nonmotor symptoms related to the disease, neuropsychiatric and cognitive manifestations, and response of symptoms to available therapies). In the second-round questionnaire, the results obtained in the first-round questionnaire were incorporated.

### 2.4. Participant Groups

To obtain valid results, the Scientific Committee estimated the minimum number of participants as 150 neurologists in the first-round, 25 in the second-round, and 125 in the third-round, to obtain a confidence level of 90% and a margin of error of ±5.35% in the first-round and a confidence level of 95% and a margin of error of ±5% in the third-round.

Participants included in the three rounds were neurologists attending more than 10 PD patients per year (66% of participants attended more than 100 patients with PD/year) and with professional activity in Spain. In the first-round, neurologists of the Movement Disorders Group of the Spanish Society of Neurology were included, while, in the second-round, neurologists with recognized expertise in PD (defined as working in specialized Movement Disorder Units), who also had participated in the first-round, were selected. Finally, neurologists of the first-round participated in the third-round again.

### 2.5. Delphi Methodology

The study was performed using a Delphi process, based on the anonymity for the individual responses, controlled opinion feedback, and statistical analysis of responses.

After fulfilling the first-round questionnaires, statistical analysis and conclusions were performed and incorporated to the second-round questionnaires, the results of which led to obtain the final conclusions, which were subsequently confirmed and assessed by the third-round experts.

According to the Delphi methodology [14, 15], consensus was established at percentages > 75% in one or two consecutive scores. Hence, symptoms were classified as (1) definitive for the diagnosis (considered as absolutely determinant by >75% of participants); (2) probable (considered as sufficient or absolute determinant by >75%); and (3) possible (considered as some, sufficiently, or absolutely determinant by >75%).

In the third-round, participants were asked about their level of agreement with the definition of APD, using the scores: 0 (no determinant), 1 (little), 2 (some), 3 (sufficiently), and 4 (absolutely).

### 2.6. Statistical Analysis

Results were expressed as a measure of central tendency and dispersion for continuous variables and as absolute numbers and relative frequencies for categorical variables. Comparisons in the clinical variables were performed using a Student's *t*-test or one-way ANOVA for continuous variables (normality assumption was previously confirmed) and Fisher exact test for categorical variables. Statistical significance was set at *p* < 0.05. The statistical analysis was performed using SAS v.9.1.3 (SAS Institute Inc., USA).

The minimum sample size (*n* = 150) was representative from the Spanish neurologists and was calculated to obtain a sample error of 0.0535, considering a maximum variability of *P* = *Q* = 50% and a confidence interval of 90%. The minimum sample size of the third-round (*n* = 125) was calculated to obtain a sample error of 0.05 at a confidence interval of 95%.

## 3. Results

### 3.1. Participants

The study was performed from May 2013 to April 2014. A total of 240 Spanish neurologists participated in the first-round and 26 in the second-round, and 149 neurologists of the first-round also participated in the third-round.

During the first-round, a total of 240 questionnaires were collected and 230 were considered valid (95.83%), from the 10 nonvalid questionnaires, 4 were incomplete, and 6 were completed by neurologists who attend less than 10 PD patients per year. Participants of the second-round attended more patients/year (65.38% attended >300 patients/year) than the first-round participants (26.52% attended >300 patients/year).

### 3.2. Determinant Factors for APD

#### 3.2.1. General Characteristics

The majority of participants considered that disease duration was a determinant factor for the diagnosis of APD (86.10% in first-round and 92.31% in second-round considered it sufficiently or absolutely determinant). Participants considered a mean disease duration of 9.17 ± 1.95 years (median = 10.00 years) as determinant factor for APD.

#### 3.2.2. Disability

In both rounds all participants considered that the type and level of disability were sufficiently (mean score = 3) or absolutely decisive (mean score = 4) for the diagnosis of APD. Second-round researchers considered that a minimum level of disability was required to establish the diagnosis of APD. 100% considered that patients have APD “when the patient requires help for the activities of daily living (such as communication, transportation, shopping, grooming, eating, or medication management)” while for 73.08% the fact that the patient has limitations to perform basic activities of daily living without help can be absolutely determinant to establish the diagnosis of APD.

#### 3.2.3. Motor Symptoms Related to Dopaminergic Treatment

The presence of motor fluctuations was sufficiently or absolutely decisive for the diagnosis of APD for 68.30% of participants in the first-round and for 88.46% in the second-round (Table 1). All participants of the second-round who were in agreement with this statement (*n* = 23) also considered that the duration of* off* periods when the best conventional treatment has been prescribed was key for the diagnosis of APD. In addition, they considered a mean percentage of waking day in* off* situation of 24.13% ± 11.45 (median: 25.00%). “Any limitation to perform basic activities of daily living, with preservation of autonomy,” and “requiring help for the activities of daily living” were also considered by 78.26% and 100%, respectively, the required level of disability during* off* periods.

88.70% of participants in the first-round and 76.92% in the second-round considered that the functional disability created by dyskinesia was sufficiently or absolutely crucial for APD diagnosis (Table 1). In the second-round, participants were asked to determine the mean percentage of daily* on* hours with dyskinesias, reporting a mean of 28.25% of the daily* on* hours with dyskinesia (median = 25.00%).

Those participants agreeing that functional disability created by dyskinesia was sufficiently or absolutely important for APD diagnosis also considered that the minimum level of disability due to dyskinesia for APD diagnosis was “requiring help for the activities of daily living” (100%) and “limitation to perform basic activities, but the patient can perform them without help” (90.00%).

#### 3.2.4. Motor and Nonmotor Symptoms Related to the Disease

For 87.80% of participants of the first-round the presence of recurrent falls was sufficiently or absolutely decisive for APD diagnosis (sufficiently: 47.40%; absolutely: 40.4%) and in the second-round 100% of participants agreed (Table 1). Freezing of gait was also considered a key factor for the diagnosis for most neurologists both in first- (82.60%) and second-round (96.15%) (Table 1). For 88.70% of neurologists in the first-round and 92.31% of participants in the second-round, alterations of postural reflexes and equilibrium were quite or absolutely determinant (Table 1). Regarding dysphagia, 92.70% in the first-round and 88.46% in the second-round considered the presence of moderate or severe dysphagia suggestive (sufficiently and absolutely determinant) of APD (Table 1). Moderate and/or severe dysarthria were considered as indicative (sufficiently and absolutely determinant) of APD for 77.40% of neurologists in the first-round and 76.92% in the second-round. The term moderate or severe dysphagia and dysarthria was defined in accordance with the UPDRS scale, part II.

In the second-round, nonmotor symptoms related to the disease such as symptomatic orthostatic hypotension (76.90%), dysautonomia (77.80%), and excessive daytime somnolence (69.20%) were some or sufficiently decisive in APD diagnosis. Although these nonmotor symptoms can be present in any stage of the disease, neurologists considered that their severity was a feature of advanced stages.

#### 3.2.5. Neuropsychiatric and Cognitive Disorders

Neuropsychiatric and cognitive manifestations of the disease such as moderate/severe depression, mild cognitive impairment, chronic presence of hallucinations with preserved insight, moderate/severe apathy, and impulse control disorders were considered as possible features for APD diagnosis (Table 1).

In the second-round 57.70% of neurologists considered moderate/severe depression as no or little determinant, 69.2% established mild cognitive impairment as some, sufficiently, or absolutely determinant, 80.80% considered hallucinations with preserved insight as some, sufficiently (26.9%), or absolutely determinant (50.0%), 34.60% agreed on moderate/severe apathy as sufficiently or absolutely determinant, and 42.20% of participants established the presence of impulse control disorders as some, sufficiently, or absolutely determinant for APD diagnosis (Table 1).

In the first-round dementia was considered sufficiently or absolutely determinant for APD diagnosis by 91.30% (sufficiently: 33.90%; absolutely: 57.40%) and by 96.15% of responders of the second-round. Hallucinations without insight were also considered as sufficiently and absolutely decisive by 83.50% of first-round respondents and by 92.31% in second-round. The psychotic symptoms were also considered decisive to some degree (some, sufficiently, and absolutely) by 91.8% and 92.31% of the first- and second-round respondents, respectively (Table 1).

#### 3.2.6. Symptoms as Indicative of APD

According to the answers obtained in the second-round and in the experience of the Scientific Committee, the following PD symptoms were considered for themselves as indicative of APD: dementia (92.31%), disability requiring help for the activities of daily living (88.46%),* off* disability (84.62%), moderate or severe dysphagia (76.92%), functional disability due to dyskinesia (76.92%), and autonomic dysfunction (53.85%) (Table 1).

#### 3.2.7. Response of APD Symptoms to Available Therapies

Regarding the degree of clinical benefit induced by different therapies on PD symptoms, 53.50% of neurologists in the first-round and 96.15% in the second-round considered that moderate/severe axial symptoms (such as balance, speech, and gait disturbances) had poor or no benefit from the available therapies. Similarly, 94.3% and 92.31% of first- and second-round respondents, respectively, also considered that dementia does not improve or poorly responds to the existing therapies. Nonmotor symptoms were considered to have moderate or no response to the available therapies by most respondents (89.10% and 92.31% in the first- and second-round, resp.). Most participants agreed that motor fluctuations and dyskinesia significantly improve with current therapies. In the first-round 91.80% of neurologists established that motor fluctuations are markedly reduced by available treatments while in the second-round the percentage reached 96.15%. Dyskinesia had moderate to excellent response to available treatments for 99.20% first-round respondents and 84.00% for second-round participants. In summary, participants considered that motor fluctuations and dyskinesia could improve with current therapies while motor symptoms related to the disease and the majority of nonmotor symptoms have a poor or absent response to the existing therapies. Then, the lack of clinical benefit with the available therapies might be considered as a factor in defining APD.

#### 3.2.8. Symptoms Classification and Final Definition

The results of the three rounds allowed us to define 3 different categories of PD symptoms that can help to define APD: definitive symptoms, probable symptoms, and possible symptoms. The symptoms were grouped in 6 different areas (general characteristics of the disease, disability, motor symptoms related to the treatment, motor symptoms related to the disease, nonmotor symptoms related to the disease, and neuropsychiatric and cognitive manifestations).

Definitive symptoms were considered those whose presence, even isolated, was enough to classify PD as APD. These included disability requiring help for the activities of daily living, presence of motor fluctuations with limitations to perform basic activities of daily living without help, severe dysphagia, recurrent falls, and dementia (Table 2).

Probable symptoms indicative of APD included time of evolution of the disease (around 10 years), limitation to perform basic activities of daily living, even if not requiring help, functional disability due to dyskinesia that covered more than 25% of waking day, moderate dysphagia, freezing of gait, moderate or severe dysarthria, and hallucinations without preserved insight (Table 2). The association of two probable symptoms of different areas made them a definite symptom.

Finally, possible symptoms included postural and balance impairment, symptomatic dysautonomia (including symptomatic orthostatic hypotension), and excessive daytime somnolence, moderate or severe apathy, chronic presence of hallucinations with preserved insight, psychotic symptoms, and mild cognitive impairment (Table 2). The combination of one possible “motor or nonmotor symptom related with the disease” areas with one possible symptom of the “neuropsychiatric and cognitive” area made them a probable symptom.

This classification of symptoms was confirmed by the majority of the neurologists of the third-round (92.00% were in complete or quite agreement), while a minority of participants was in slight (7.38%) or poor agreement (0.67%). Therefore, based on these results, APD can be defined as an advanced stage of PD in which certain symptoms and complications are present, with a detrimental influence on the overall patient's health conditions and with a poor response to conventional treatments (Table 2).

## 4. Discussion

Current evidence suggests that both motor and nonmotor symptoms significantly contribute to health status and quality of life in PD [8].

A progressive limitation for carrying out the usual activities of daily living (disability) with disease progression is very common in PD. Such limitation results from a combination of motor impairment and complications, but also from nonmotor symptoms that can restrict the activity through a diversity of ways (fatigue, apathy, cognitive impairment, pain, etc.) and aging [16, 17]. As the disability increases, patients require to be helped for carrying out basic activities of daily living and, finally, are totally dependent of caring by others, leading to a great negative impact on patients and caregivers life [18]. Therefore, disability must be considered a fundamental feature for grading PD stage, although other characteristics as the disease duration, usually longer than 10 years, or the incapacity to obtain enough clinical benefit from conventional therapies are also important in defining APD.

Today, however, there is no agreement among neurologists concerning the clinical features that PD should exhibit to be considered as APD [4, 5].

Using the Delphi methodology in a large sample of neurologists we have obtained a consensus on the definition and clinical characteristics that PD patients exhibit in the advanced stage of PD. Most importantly, we have been able here to describe some definitive symptoms of PD that, when present, are sufficient to classify patients as having APD. In addition, this study has allowed us to identify certain symptoms that, when combined, could allow identifying possible and probable APD patients.

We found that both motor and nonmotor symptoms were definitive for the diagnosis of APD, either related to the evolution of the disease or related to the long-term levodopa therapy. In fact, the appearance of certain intrinsic motor symptoms of PD, as recurrent falls and severe dysphagia were definitive determinants for the diagnosis of APD, while moderate dysphagia and freezing of gait were probable symptoms for APD diagnosis. Classically, these manifestations have been associated with advanced disease and, frequently, parallel the appearance of cognitive decline [19, 20].

The development of severe motor fluctuations with disabling “off” periods was also considered a definitive factor for APD diagnosis. These motor complications can appear early in the course of the disease, particularly in young patients, and rethought to reflect the extent of nigrostriatal degeneration. These motor manifestations can dramatically improve with currently available therapies like DBS, continuous subcutaneous apomorphine infusion, or the infusion of levodopa/carbidopa intestinal gel.

Accordingly, we can suggest that APD would be reached when the underlying pathological substrate of PD is widespread but also when PD patients develop the characteristic complications associated with long-term levodopa treatment [20, 21]. In both instances the common feature is the impact they have on disability and quality of life. In fact, in our study the disability created either by the disease or by long-term levodopa therapy was considered for 100% of participants as a definitive factor for APD diagnosis.

Neuropsychiatric manifestations are common in both nondemented and demented PD patients [22]. Most participants established that dementia and hallucinations without insight are, respectively, definitive and probable symptoms for APD diagnosis. This is consistent with studies showing that more than 80% of PD patients develop dementia after 20 years of the disease evolution and also with reports describing that hallucinations are probably the clinical symptom most consistently associated with progressive cognitive deterioration and dementia in PD [23].

The number and severity of the nonmotor symptoms, as a whole, increase with PD progression, although in a variable manner [24–26]. Nonetheless, in late stages of the disease, the nonmotor symptoms may be the dominant problem in many patients [27] and have a huge impact on patient's health state, quality of life, and instrumental functionality [28–30].

Finally, in order to assess the real impact of this consensus in clinical practice we should now evaluate whether or not the 3 different categories of symptoms (definitive, probable, and possible) ascribed to APD clearly classify PD patients in different stages of the disease in the clinical practice.

## Figures and Tables

**Table 1:** Percentages of respondents in both rounds.

Symptoms	% of participants					
	First-round		Second-round			
	Sufficiently and absolutely	Some, sufficiently, and absolutely	Absolutely	Sufficiently and absolutely	Some, sufficiently, and absolutely	Indicative symptoms of APD
(A) Motor symptoms related to treatment						
Motor fluctuations	68.30			88.46		
Duration of *off* periods > 25%	89.50		100.00			
*Off* disability	92.10		100.00			84.62
Limitation to perform instrumental activities			47.83			
Limitation to perform basic activities (but without help)			78.26			
Requiring help for daily living activities			100.00			
Durations of *on* dyskinesias > 25%	53.50					
Functional disability due to dyskinesias	88.70			76.92		76.92
Limitation to perform instrumental activities			50.00			
Limitation to perform basic activities (but without help)			90.00			
Requiring help for daily living activities			100.00			

(B) Motor symptoms related to the disease						
Recurrent falls	87.80			100.00		
Freezing of gait	82.60			96.15		
Alteration of postural reflexes and equilibrium	88.70			92.31		
Moderate or severe dysphagia	92.70			88.46		76.92
Moderate or severe dysarthria	77.40			76.92		

(C) Neuropsychiatric and cognitive disorders						
Moderate/severe depression	20.50	57.50		15.40	42.30	
Mild cognitive impairment	29.50	76.50		26.90	69.20	
Dementia	91.30	97.00		96.15		92.31
Chronic presence of hallucinations with preserved insight	39.50	83.80		50.00	80.80	
Hallucinations without insight	83.50	95.70		92.31		
Psychotic symptoms	73.50	91.80			92.31	
Moderate/severe apathy	45.60	76.50		34.60	69.20	
Impulse control disorders	29.10	65.20		38.40	42.20	

**Table 2:** Results obtained in the third-round.

Level of relevance	General characteristics	Disability	Motor symptoms related with treatment	Motor symptoms related with the disease	Nonmotor symptoms related with the disease	Neuropsychiatric and cognitive symptoms
Definitive symptoms		Requiring help to perform daily living activities	Presence of motor fluctuations with an *off* time > 25%, with limitation to perform basic activities, without requiring help	Severe dysphagia Recurrent falls		Dementia

Probable^*∗*^symptoms	Evolution time (around 10 years)	Limitation to perform basic activities, although not requiring help	Functional disability due to dyskinesias with an *on* time > 25%	Moderate dysphagia Freezing of gait Moderate-severe dysarthria		Hallucinations without preserved insight

Possible^*∗∗*^ symptoms				Postural and equilibrium disorders	Symptomatic dysautonomia, including orthostatic symptomatic hypotension, excessive daytime somnolence	Moderate-severe apathy Chronic presence of hallucinations with preserved insight Psychotic symptoms Mild cognitive impairment

^*∗*^The association of two probable symptoms of different areas (general characteristics, disability, motor symptoms related to treatment, etc.) makes them a definite symptom.

^*∗∗*^The association of one possible motor or nonmotor symptom related with the disease areas with one possible symptom of the neuropsychiatric and cognitive area makes them a probable symptom.
